# Post‐Cholecystectomy Mirizzi Syndrome by Calculus in the Remaining Cystic Duct: Case Report and Review of Literature

**DOI:** 10.1155/cris/6658220

**Published:** 2026-06-29

**Authors:** Jorge López-López, Luis García-Covarrubias, Selene Y. Flores de León, Diana F. Fernández-Ángel, David Ramírez-Tapia, José H. Garza-Flores, Vicente González-Ruíz

**Affiliations:** ^1^ Surgery Department, Hospital General de México Dr. Eduardo Licega, Mexico City, Mexico; ^2^ Gastro-Surgery Department, Specialties Hospital, CMN SXXI, IMSS, Mexico City, Mexico, imss.gob.mx

**Keywords:** biliary obstruction, case report, cystic duct, gallbladder remnant

## Abstract

**Background:**

Mirizzi Syndrome (MS) is a rare cause of biliary obstruction. While laparoscopic cholecystectomy is the standard treatment, severe inflammation often necessitates subtotal cholecystectomy to prevent bile duct injury (BDI). However, retained stones in the gallbladder remnant or cystic duct can cause recurrent obstruction, termed post‐cholecystectomy Mirizzi Syndrome (PCMS).

**Case Report:**

A 34‐year‐old female presented with PCMS 6 years after undergoing a subtotal cholecystectomy for MS. Despite initial conservative management, definitive resolution required secondary surgical intervention.

**Conclusion:**

As subtotal cholecystectomy becomes more frequent to avoid BDI, the incidence of PCMS is expected to rise. Clinicians must maintain a high index of suspicion. Management should be multidisciplinary; however, surgery remains the definitive treatment when conservative approaches fail.

## 1. Background

Laparoscopic cholecystectomy is the definitive treatment for symptomatic gallstones. However, chronic inflammation and anatomical distortion often increase the risk of iatrogenic bile duct injury (BDI). In such high‐risk scenarios, subtotal or partial cholecystectomy is a safe, widely accepted alternative [1–3].

Despite its safety profile, this approach can lead to late complications. An inflammatory process in the gallbladder remnant or extrinsic compression of the common hepatic duct by stones in the cystic duct stump defines post‐cholecystectomy Mirizzi syndrome (PCMS) [4, 5]. This rare entity remains a diagnostic and therapeutic challenge [5].

## 2. Case Presentation

A 34‐year‐old female presented with a 6‐day history of sudden, severe epigastric pain (intensity 10/10 on the visual analog scale) radiating to the right hypochondrium. Associated symptoms included jaundice, nausea, emesis, choluria, and acholia; she denied experiencing fever or chills.

Her surgical history was notable for a laparoscopic cholecystectomy performed 6 years prior for acute cholecystitis. Operative findings at that time had revealed a high‐tension gallbladder measuring 17 × 7 × 8 cm, an 8‐mm gallbladder wall, and a 22‐mm stone impacted in Hartmann’s pouch, which was classified as Mirizzi syndrome (MS) type I. She was discharged on postoperative day 3 following an uneventful recovery.

Upon readmission, physical examination revealed overt clinical jaundice and epigastric tenderness without signs of peritonitis. Laboratory evaluation demonstrated marked cholestasis and hepatocellular injury: total bilirubin 7.64 mg/dL, direct bilirubin 4.0 mg/dL, gamma‐glutamyl transferase (GGT) 875 U/L, alkaline phosphatase (ALP) 281 U/L, aspartate aminotransferase (AST) 406 U/L, and alanine aminotransferase (ALT) 640 U/L. Hematological and coagulation profiles were within normal limits.

Magnetic resonance cholangiopancreatography (MRCP) demonstrated a common bile duct (CBD) dilated to 14 mm, with a 9.5‐mm filling defect within its pancreatic segment, accompanied by a tortuous and dilated cystic duct remnant measuring 10.2 mm (Figures 1 and 2).

**Figure 1:**
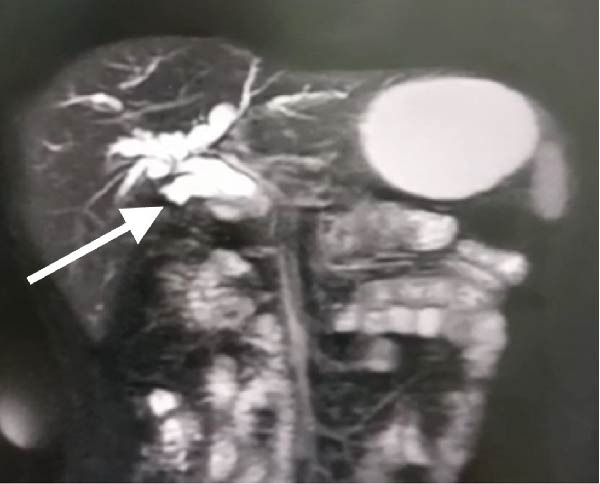
Magnetic resonance cholangiopancreatography (MRCP) demonstrating a dilated common bile duct measuring 9 mm, proximal to a faceted signal defect consistent with a 4 × 6 × 9 mm calculus (arrow). Compression of the distal bile duct is also noted, likely secondary to the impacted gallstone.

**Figure 2:**
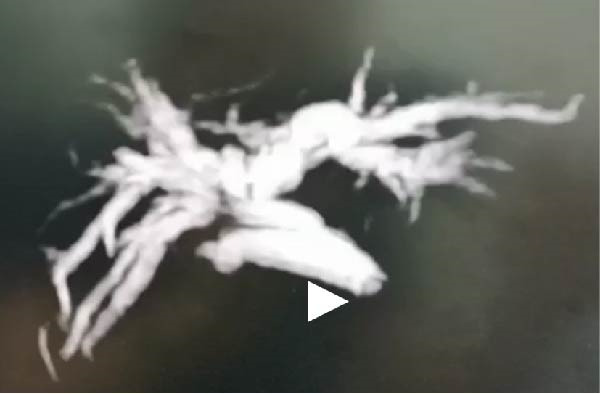
Magnetic resonance cholangiopancreatography (MRCP) reconstruction demonstrating a long and dilated cystic duct remnant with a characteristic “silkworm” appearance. A filling defect is observed at its distal end (arrowhead), with contrast opacification extending near its insertion at the junction of the common hepatic duct and the common bile duct.

Subsequent endoscopic retrograde cholangiopancreatography (ERCP) identified extrinsic compression of the middle third of the CBD caused by an impacted calculus within the cystic duct stump Endoscopic retrieval of the stone was unsuccessful (Figure 3).

**Figure 3:**
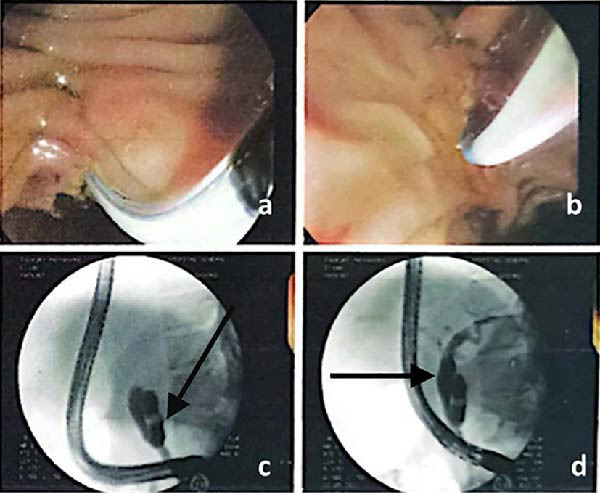
Endoscopic retrograde cholangiopancreatography (ERCP). (a) Technical difficulty, with six attempts required for bile duct cannulation. (b) Successful cannulation of the bile duct after more than 5 min. (c) Dilated cystic duct remnant (arrow), measuring up to 8 mm in diameter and containing two filling defects (4 × 6 mm and 6 × 9 mm), consistent with gallstones, producing extrinsic compression of the common hepatic duct. (d) Normal‐appearing intrahepatic ducts and a common bile duct dilated to 8 mm.

The patient underwent surgical exploration 1 week later. Adhesiolysis revealed a 6.5 × 1.5 cm cystic duct remnant retaining surgical staples and containing purulent bile. A 5‐mm stone was extracted from this remnant. Following a longitudinal choledochotomy, multiple small calculi were retrieved from the common hepatic duct, which was dilated to 12 mm. A T‐tube was placed for biliary decompression, and intraoperative cholangiography confirmed adequate contrast clearance and biliary patency into the duodenum (Figure 4).

**Figure 4:**
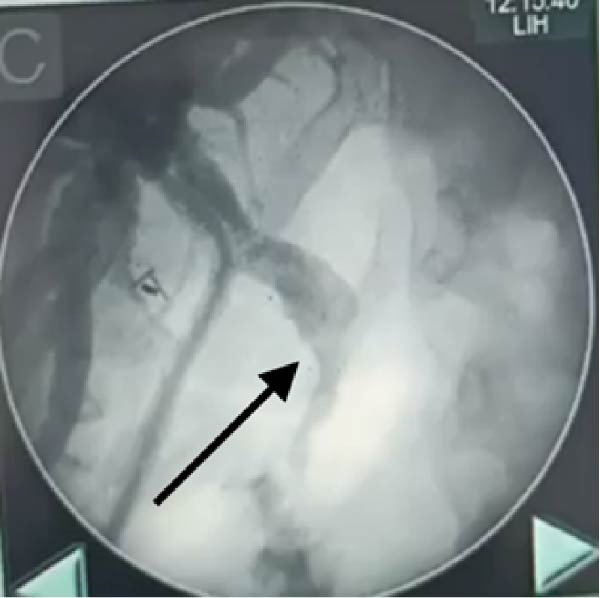
Intraoperative cholangiography showing no evidence of filling defects (arrow). The patient was discharged on postoperative day 3 without complications.

The T‐tube was removed 6 weeks after a normal follow‐up cholangiogram (Figure 5).

**Figure 5:**
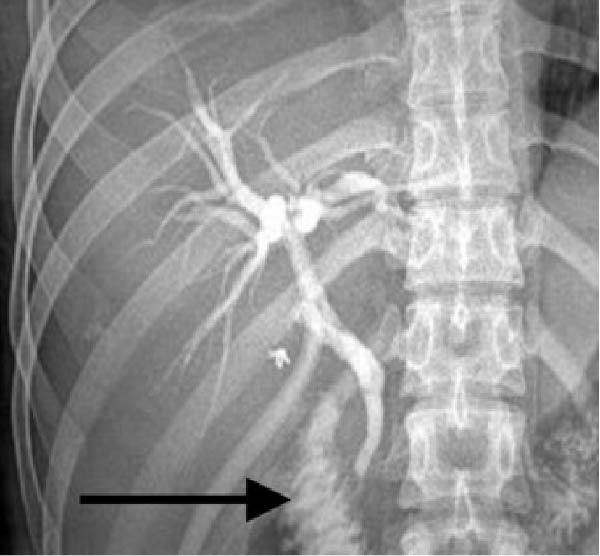
T‐tube cholangiography performed 6 weeks after surgery, showing no evidence of residual choledocholithiasis and demonstrating rapid drainage of contrast medium into the duodenum.

## 3. Discussion

Post‐cholecystectomy syndrome (PCS) remains a clinically relevant challenge despite the high efficacy of cholecystectomy, which successfully resolves gallstone‐related symptoms in up to 85% of cases. However, between 5% and 47% of patients experience persistent or recurrent gastrointestinal symptoms after surgery—a phenomenon first described by Womack and Crider in 1947. This wide variation in reported incidence reflects the heterogeneous and multifactorial nature of the syndrome [6].

The etiology of PCS is complex, encompassing biliary, extra‐biliary, and functional causes. Among these, biliary mechanisms are critical because they are frequently amenable to targeted intervention. Epidemiologically, PCS shows a female predominance, with a reported male‐to‐female ratio of 1:1.45 [7]. Additionally, the severity and duration of preoperative symptoms have been consistently identified as major risk factors [8–11]. One of the primary biliary causes of PCS is a cystic duct remnant or gallbladder stump. First characterized by Florcken in 1912 as “cystic duct remnant syndrome,” this entity involves a residual cystic duct measuring more than 1 cm post‐cholecystectomy [12, 13]. These remnants can harbor retained or recurrent calculi and act as a focus for chronic inflammation, driving recurrent symptoms. Cystic duct stump stones are a well‐recognized trigger for PCS, accounting for a significant proportion of patients presenting with post‐cholecystectomy abdominal pain or obstructive jaundice [12–14].

Within this clinical spectrum, PCMS represents a rare but severe complication resulting from extrinsic compression of the CBD by stones impacted within the cystic duct remnant or gallbladder stump [14]. Affected patients typically present with biliary colic, acute cholangitis, pancreatitis, or obstructive jaundice [14]. Documented risk factors include prior subtotal cholecystectomy, a long cystic duct remnant (>1 cm), and anatomical variants such as a low insertion or a parallel course of the cystic duct relative to the common hepatic duct [14]. In our patient, the cystic duct anatomy corresponded to type IV of the Hahn and Blumgart classification, a configuration known to predispose to stone impaction and subsequent biliary obstruction [14] (Figures 1 and 2).

Diagnosing PCMS remains challenging and demands a high index of suspicion. While abdominal ultrasound is standard for the initial evaluation, its sensitivity is limited [15]. ERCP remains the diagnostic gold standard due to its combined diagnostic and therapeutic capabilities. Magnetic resonance cholangiopancreatography (MRCP) provides a valuable, non‐invasive alternative that clearly delineates extrinsic bile duct compression and associated findings, such as cystic duct dilation proximal to an obstructing stone [16]. In certain instances, this produces the characteristic “silkworm sign” observed in our patient. Computed tomography offers limited diagnostic utility for the biliary tree itself but remains useful for excluding malignant etiologies [16–18].

Several classification systems categorize Mirizzi syndrome and its variants. The Csendes classification stratifies disease severity based on the degree of bile duct erosion and the presence of a cholecystobiliary fistula. More recently, PCMS has been classified as a distinct subtype (type Ib), differentiating it from classic Mirizzi syndrome by the patient’s history of prior cholecystectomy. PCMS can be further subclassified into early or late onset based on the timeline of postoperative presentation; late cases are more frequently tied to recurrent lithiasis, secondary biliary strictures, or sphincter of Oddi dysfunction [19, 20].

Management of PCMS must be individualized. Initial stabilization requires aggressive intravenous fluid resuscitation, particularly in patients presenting with systemic inflammatory response or sepsis [20]. Minimally invasive approaches are preferred as first‐line therapy, reserving open surgery for refractory or anatomically complex cases [20, 21]. ERCP is widely utilized to achieve biliary decompression and stone extraction. However, technical difficulties in cannulating a tortuous cystic duct remnant or removing deeply impacted stones can compromise its success [21]. Consequently, some authors advocate for ERCP with sphincterotomy and temporary biliary stenting as a temporizing measure, followed by definitive surgical resection of the gallbladder remnant [21].

Advances in advanced endoscopy have expanded the therapeutic arsenal. Intraductal lithotripsy—including electrohydraulic lithotripsy (EHL) and cholangioscopy‐assisted laser lithotripsy (CA‐LL)—allows for the fragmentation of difficult stones under direct visualization [22]. Platforms such as the SpyGlass system enable precise targeting and higher clearance rates [22, 23]. Despite their clinical utility, these techniques demand specialized expertise and are not universally available [22, 23]. Single‐operator cholangioscopy (SOC) facilitates both the diagnosis and management of complex biliary stones [23]. Following lithotripsy, a meticulous cholangioscopic inspection is essential to confirm complete clearance of the cystic duct and main biliary channel. Nonetheless, limited availability and technical complexity restricted its use in our center, as in the present case [23].

Surgical management remains a cornerstone of treatment when endoscopic options fail or are anatomically unfeasible. Although open completion cholecystectomy was historically preferred due to dense postoperative adhesions, modern laparoscopic techniques have made minimally invasive completion cholecystectomy increasingly feasible in specialized centers [24]. Nonetheless, revisional surgery carries inherent risks of hemorrhage, iatrogenic biliary injury, and wound complications [24].

The laparoscopic‐endoscopic rendezvous (LERV) technique represents a hybrid approach designed to facilitate bile duct cannulation and mitigate the risk of post‐ERCP pancreatitis [25]. This procedure involves the antegrade placement of a transcystic guidewire during laparoscopy, which is then retrieved endoscopically to guide retrograde cannulation and stone extraction in a single stage. LERV has been associated with favorable outcomes, including lower complication rates and reduced hospital stays [25]. However, it is technically demanding and requires seamless coordination between the surgical and endoscopic teams, limiting its adoption in resource‐constrained environments. Additionally, it may be unsuitable in the setting of large impacted stones, severe biliary strictures, or periampullary diverticula. To date, current evidence has not definitively established the superiority of LERV over conventional two‐stage management [25].

Ultimately, PCMS is an uncommon but critical etiology of PCS that requires prompt recognition. Advanced imaging is central to the diagnostic workup, and definitive treatment must be tailored to the patient’s clinical status, local biliary anatomy, and institutional expertise. While innovations in endoscopic and minimally invasive surgical modalities have significantly optimized outcomes, the management of complex cases remains highly challenging.

## 4. Conclusions

PCMS is an important postoperative biliary complication following cholecystectomy, particularly after subtotal procedures. It should be considered in post‐cholecystectomy patients presenting signs of bile duct obstruction. Endoscopic and percutaneous approaches may be effective in selected cases; however, surgical management remains necessary when minimally invasive options are unsuccessful or unavailable.

## Funding

The authors state that the research and publication of their article did not receive specific funding and was performed as part of the employment of the authors in the Hospital General de México “Dr. Eduardo Liceaga.”

## Disclosure

The publication consent letter cannot be uploaded or treated as supporting information according to journal requirements.

## Consent

Written informed consent was explicitly obtained from the patient for the publication of this case report and any accompanying images. A copy of the written consent is available for review by the editor‐in‐chief of this journal.

## Conflicts of Interest

The authors declare no conflicts of interest.

## Data Availability

The data that support the findings of this study are available upon request from the corresponding author. The data are not publicly available due to privacy or ethical restrictions.
